# Improved assessment of mass drug administration and health district management performance to eliminate lymphatic filariasis

**DOI:** 10.1371/journal.pntd.0007337

**Published:** 2019-07-05

**Authors:** Carmen Maroto-Camino, Pilar Hernandez-Pastor, Naomi Awaca, Lebon Safari, Janet Hemingway, Marilia Massangaie, Donald Whitson, Caroline Jeffery, Joseph J. Valadez

**Affiliations:** 1 Department of International Health, Liverpool School of Tropical Medicine, Liverpool, United Kingdom; 2 National NTD Programme, Democratic Republic of Congo, Ministère de la Sante Publique, Gombe, Kinshasa; 3 National NTD Programme, Direção Nacional da Saúde Publica, Ministério da Saúde, Eduardo Modliane, Maputo, Mozambique; University Hospital Bonn, GERMANY

## Abstract

Lymphatic filariasis (LF) elimination as a public health problem requires the interruption of transmission by administration of preventive mass drug administration (MDA) to the eligible population living in endemic districts. Suboptimal MDA coverage leads to persistent parasite transmission with consequential infection, disease and disability, and the need for continuing MDA rounds, requiring considerable investment. Routine coverage reports must be verified in each MDA implementation unit (IU) due to incorrect denominators and numerators used to calculate coverage estimates with administrative data. IU are usually the health districts. Coverage is verified so IU teams can evaluate their outreach and take appropriate action to improve performance. Mozambique and the Democratic Republic of Congo (DRC) have conducted MDA campaigns for LF since 2009 and 2014, respectively. To verify district reports and assess the declared achievement using administrative data of the minimum 80% coverage of eligible people (or 65% of the total population), both countries conducted rapid probability surveys using Lot Quality Assurance Sampling (LQAS)(n = 1102) in 2015 and 2016 in 58 IU in 49 districts. The surveys identified IU with suboptimal coverage, reasons residents did not take the medication, place where the medication was received, information sources, and knowledge about diseases prevented by the MDA. LQAS identified four inadequately covered IU triggering district team performance reviews with provincial and national teams and district retreatment. Provincial estimates using probability samples (weighted by populations sizes) were 10 and 17 percentage points lower than reported coverage in DRC and Mozambique. The surveys identified: absence from home during annual MDA rounds as the main reason for low performance and provided valuable information about pre-campaign and campaign activities resulting in improved strategies and continued progress towards elimination of LF and co-endemic Neglected Tropical Diseases.

## Author summary

Global elimination of lymphatic filariasis (LF) is achieved through treatment of at-risk populations with annual or bi-annual mass drug administration campaigns. In Africa campaigns need to be completed in 32 countries with 343 million people at risk. The World Health Organisation recommends verification of the campaign’s administrative records using household cluster surveys at least once every 5-years. However, cluster surveys are expensive and usually completed in a few districts only or at sub-national levels. Together with National Programmes in Mozambique and the Democratic Republic of the Congo (DRC) we adapted Lot Quality Assurance Sampling (LQAS) methods to verify campaign coverage because it is relatively inexpensive in comparison to other survey approaches, provides information and facilitates actions at the Implementation Unit (IU) level, which is usually the district. LQAS signals IU whose performance is likely to need improvement because MDA coverage is below the coverage target. Our results show consistently that administrative records over-estimated campaign coverage and did not detect implementation and coverage problems due to errors in numerators and denominators, incorrect reporting, and/or incorrect aggregation of tally sheets. The LQAS verification approach prompted immediate action to remedy coverage shortfalls averting persistent LF transmission and disease, and the costs associated with failed campaigns. Our study demonstrates that a rapid probability sample to verify coverage provides district teams with information after each campaign which can be used for action, and that one coverage survey every 5-years is insufficient for infectious disease elimination in highly endemic settings where achievement of optimal coverage is essential. It also demonstrates that LQAS provides a decentralised assessment, sensitive for detecting and ameliorating programme bottlenecks and can be used to verify MDA in other countries.

## Introduction

Five Neglected Tropical Diseases (NTDs), lymphatic filariasis (LF), onchocerciasis, schistosomiasis, trachoma and soil transmitted helminths, have been prioritised by the World Health Organisation (WHO) for elimination as public health problems by 2020. They were chosen due to their high prevalence, resulting morbidity and disability and the susceptibility of the parasites, causing the diseases, to efficient preventive treatment through mass drug administration (MDA) [1].

Lymphatic Filariasis is endemic in 32 countries in Africa with a population of more than 343 million people [2] living at risk. The infection is acquired in childhood. The adult parasite causes severe damage to the lymphatic system, which in 30% of the infected people, results in long-term disability [3]. Estimates of the morbidity caused by LF are complex due to stigma associated with the disease.

Despite a suspected reduction in LF prevalence due to MDA, in 2013 there were 19.43 million cases of hydrocele and 14.41 million cases of Lymphedema attributed to the parasite Wuchereria Bancrofti, which is more prevalent in Africa [4]. LF is, according to the WHO, the second leading cause of long-term disability with an estimated 2.78 to 5.09 million DALYs lost per year [5]; in recent review of the Global Burden of Diseases estimated that 25 million DALYs were lost in 2015 despite the elimination activities[6].

Adult worms of *Wuchereria bancrofti*, live in people for five to 8-years, producing millions of larvae (microfilaria), which after undergoing transformation in mosquitoes, are transmitted to other people. The mainstay of the elimination campaign is the destruction of the microfilaria by the use of preventive medication. In African countries LF is frequently co-endemic with Onchocerciasis. In these countries the recommended elimination strategy is annual treatment of the eligible population (population living in the endemic districts over 5-years of age) with one dose of Ivermectine and Albendazole. In areas in Western Africa with co-endemic Loa-loa, and the possibility of serious encephalopathy when Ivermectine is administered[7], WHO recommended treatment with Albendazole every six months for five consecutive years.

Elimination of LF as a public health problem requires a minimum of five consecutive annual MDA rounds with effective coverage, which is defined by the WHO as administration of medication to at least 65% of the total population. As the eligible population is the population over 5-years of age, at least 80% coverage of the eligible population needs to be reached (80% coverage x 80% of population is eligible = 64% total population rounded to 65% [1, 8]. As reaching optimal coverage is the key to LF elimination [9], we use 80% medication coverage of the eligible population as the minimum target to be achieved by all IU. We recognize high transmission settings may require higher coverage because of the reservoir of parasites in the population not taking medication or who are not eligible for it, and the persistence of microfilaremia in populations not treated [10, 11] due to co-endemicities.

Reported coverage typically is the number of people registered as treated during the campaign (numerator) divided by population estimate based on a recent census (as is the case in Mozambique) or by a census of houses prior to the start of an MDA campaign (as in DRC). Both methods can produce errors both in the numerator and the denominator.

In Mozambique medication is administered in “concentrações” or distribution points by mobile and fixed teams with a daily district target of 600–1500 persons based on estimations of the National Institute of Statistics. Administrative data is collected (age, gender and number of pills provided to each person) by one team member (usually the community health worker), as the treatment proceeds. It is then aggregated at the end of each distribution day and handed to the district data manager in a “daily reporting form.” Sometimes these forms are compared with other data sources showing medication consumption. These daily distribution data are added to the campaign database and presented to the district management team. District data is sent to the province and national programmes. Campaigns are 5-days. At their end the district team send a final distribution report to provincial and national managers along with a summary of the medication received and administered.

In DRC, medication is administered house-to-house by volunteer Community Drug Distributors (CDDs), who treat people in 20 houses (in towns this number increases to 100 houses); they are supervised by the “Infirmier titulaire” of the nearest health care centre. Although they complete a census of the population before the campaign, they may under-report “registration” and over-report coverage to avoid going back to houses with absent residents.

In DRC the campaign follows the community-based Ivermectine distribution strategy in which each community conducts their campaign at their own time, which means that despite efforts to shorten the time, campaigns can take as long as 3-months to complete in one province. CDD records pass sequentially to sub-districts, districts, provinces and finally to the central level. This process can take several months as data are aggregated, cleaned and analysed at each level.

While effort has gone into improving routine health information systems, and the hope is that they will eventually provide more accurate health information, several studies demonstrate that they remain inaccurate[12, 13]. Recognising these short comings, WHO has requested that endemic countries conduct household surveys to validate the reported MDA coverage rate and support remedial action where microfilaremia persists despite reports of achieved coverage targets. Our literature search of LF elimination activity indicates that household survey data are scarce; and often only presented in annual meetings [14]. One report suggests using community monitoring to validate reported national coverage [15]. The lack of capacity and verified results at the IU level (typically districts) are major obstacles to obtaining useful results for improvement.

Mozambique and the DRC are both endemic for LF. Mozambique has 115 districts endemic for LF and due to co-endemicity with Onchocerciasis has undertaken MDA with Ivermectin (IVM) and Albendazole (ALB) since 2009. DRC has 245 LF endemic districts and started MDA in 2014 with IVM and ALB for co-endemic LF-Onchocerciasis districts, and in 2015 with ALB alone twice a year in Kongo Central Loa-LF co-endemic areas [16]. We worked with the National NTD programmes in Mozambique and DRC to adapt Lot Quality Assurance Sampling (LQAS) methods to verify MDA coverage at the district and sub-district level to rapidly detect sub-optimal coverage, and provide relevant information to the IU teams to improve strategies for distribution in their areas of operation. LQAS was originally developed to assess industrial batch production in the 1920s[17–19], and subsequently adapted in the mid-1980s to improve primary health care services[20, 21], This study demonstrates its utility for LF programme improvement, and exhibits the error present in administrative data.

## Methods

*Ethical approvals*: This study was reviewed and approved by the National NTD Programmes of the Ministries of Health of the Democratic Republic of Congo and the Republic of Mozambique.

*LQAS principles*: Industrial LQAS takes a sample (n) of items from a lot (batch of goods) to assess if it has not reached a production standard. If the number of functioning items in the sample does not reach a statistically predetermined cut-off value (d), the lot is classified as failing to reach the production standard. This decision rule is set using cumulative probabilities of binomials and optimises the identification of very low performance areas.

We applied LQAS principles to MDA coverage. The cut-off value (d) was dependant on the sample size, the coverage target (p-upper or p_U_) and very low (p-lower or p_L_) covered areas that signified the urgent need for improvement, and the selection of two misclassification errors—the probability of misclassifying an area with high coverage (≥p_U_) as low (α error) and the probability of misclassifying an area with very low coverage (≤ p_L_) as high (β error). Areas with intermediate performance (>p_L_ but < p_U_) are classified as high or low depending on their proximity to p_L_ or p_U_.

*Study Area and Population*: In Mozambique, we surveyed MDA coverage in 21 Supervision areas of 18 districts of four provinces (Cabo Delgado, Nampula, Niassa and Zambezia) in 2016 (n = 399) (Table 1). The population data used by districts for planning activities were provided by the National Institute of Statistics and consisted of projections from the 2007 national census. Districts were randomly selected among districts in their 5^th^ campaign. Three districts were divided into two supervision areas (Cuamba, Inhassunge, Mueda) due to their large size or logistical complexity.

**Table 1:** Study characteristics in Mozambique and DRC for 2015–2016.

Country	Date	Drug Administered	Provinces (Catchment Areas)	Total number of Supervision Areas (districts)	Total Sample Size
Mozambique	2016	Ivermectin and Albendazole	Cabo Delgado, (Nampula, Niassa and Zambézia)	21 (18)	399
DRC	2015	Albendazole	Kongo Central (co-endemic Loa-LF districts)	9 (9)	171
DRC	2016	Albendazole	Kongo Central (co-endemic Loa-LF districts)	10 (9)	190
DRC	2016	Ivermectin and Albendazole	Kasai, Kasai Central, Kasai Oriental (co-endemic Oncho-LF districts)	18 (13)	342

In the DRC, we surveyed ALB treatment in nine Loa-LF districts in the Kongo Central province in 2015 (n = 171) and 2016 (n = 190) with each district being a supervision area in 2015 (during the first MDA) and a subdivision of Kuimba district in 2016 to assess performance of sub-districts in their first campaign while all other districts were in their second round. In 2016, we surveyed 13 additional districts in the three provinces of Kasai. The districts were randomly selected among all LF endemic districts in the provinces, completing their second MDA. The districts were subdivided in 18 supervision areas to take account of population and rural-urban differences. In these provinces (Kasai, Kasai Central and Kasai Oriental), we measured IVM and ALB coverage (n = 342) (Table 1).

**Fig 1:**
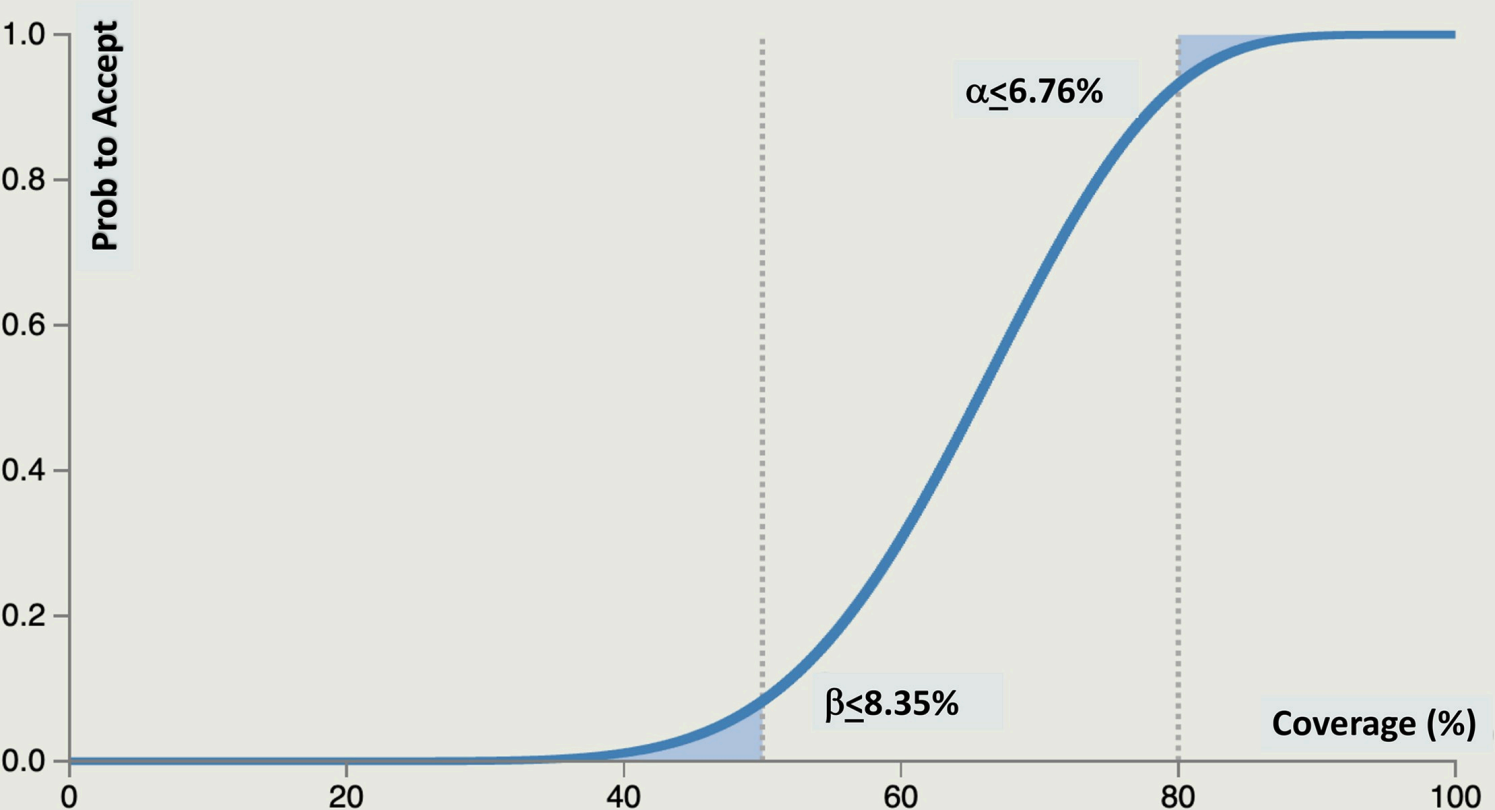
Operating characteristic curve for p_U_ = 0.80, p_L_ = 0.50, d = 13 showing the probability of being classified as reaching p_U_.

For both countries, p_U_ = 80%, while p_L_ = 50%. The resulting parameters for classifying each implementation unit are: n = 19, d = 13, α = 0.068 and β = 0.084. LQAS error is displayed explicitly with an Operating Characteristic Curve (OCC) (Fig 1) showing the probability an implementation unit (called *supervision areas* (SAs)) with any level of coverage is classified as reaching p_U_. Districts closer to either threshold (p_U_ or p_L_) are more likely to be classified in that category. LQAS is set to identify the worst performer districts. When multiple SA are aggregated, they are treated as a stratified random sample to calculate coverage with 95% confidence.

In each SA we surveyed 19 locations selected with probability proportional to size. In the selected location (village or area of towns), a household was randomly selected using segmentation sampling [22]; for this process communities were divided into segments containing approximately the same number of households. A segment was then chosen randomly; households were enumerated, and one was sampled randomly. Eligible people in the household were listed, and one was selected randomly for interview. Data collectors use random number tables in the field for random selection.

Following the classification, the SAs were aggregated as a stratified random sample to measure coverage in the provincial Catchment Area. See Table 1 for a description of the samples in each country, the drugs administered with dates, provinces, the number of SA classifications, and the total sample sizes.

In the DRC, CDDs conduct a census of the eligible population before an MDA, through door-to-door registration, to determine the denominator and obtain appropriate numbers of donated drugs. Registration information included: number of eligible people in each household, and their age, gender, house number, community (or neighbourhood), reasons for not taking the medication, knowledge about diseases prevented by it, the information source about the campaign and the CDD’s name. The main indicator was whether all eligible people were registered and took the appropriate medication during the annual MDA campaign.

### Data collection

In Mozambique, two-weeks after the MDA, 21 data collectors and nine supervisors were trained for four days prior to surveying 21 SA in four provinces during 1–9 July 2016. In the DRC, 3 supervisors and 9 surveyors for Kongo Central were trained in Matadi in 2015. In 2016 40 data collectors and 10 supervisors were trained shortly after the MDA in five locations after training of trainers in Kinshasa from 14–17 July 2016 to facilitate this roll out, then replicated in the three capitals of the Kasai provinces (Kananga, Tshikapa and Mbuji-Maji) and in Matadi for Kongo Central. Data collection occurred immediately following the training. LSTM supervisors were deployed in each province to provide support to the national and provincial supervisors who verified the selection process and the interviews before the questionnaire was sent to the database.

Data were collected using the following protocol. The sampling frame consisted of the list of villages with their population size supplied by the National Programs. Villages were selected using probability proportional to size. Upon arriving at the village, the enumerator visited the community leader to explain the purpose of the survey. With his help they constructed a hand drawn map of the community which was divided into segments of approximately the same size. One segment was selected randomly with a random number table [22]. The enumerator constructed a map of the segment and listed the houses, one of which was selected randomly and used as a reference house. In order to reduce the likelihood that a house was not included in the map and had zero probability of selection, the next nearest house walking distance to the door of the reference house became the starting point for the first interview. A list of eligible people was constructed and one person randomly selected with a random number table. If the person was present and gave consent the interview was completed. If the person was ≤12 years of age but ≥5-years, the mother or the father responded for the child, but in the presence of the child. If the selected person was not present but was in the village, the enumerator sent someone to bring him/her to the house. If the selected person was not in the village but returning in about 30 minutes the enumerator waited for him/her. If the person was not returning within 30 minutes and a family member had seen him/her take the medication then the family member responded. If it was possible for the enumerator to contact her/him by phone then the responses were corroborated. The enumerator returned in the evening to the home in the case the person was returning at night and information had not yet been corroborated.

If the person was not returning and not able to be contacted then that person was not included in the sample. The house with the closest door to the current house’s front door was selected and the selection process for an eligible respondent was restarted.

In Mozambique and DRC rural areas daily agricultural work begins at sunrise and by about 10AM most family members return to their homes. In urban areas or towns eligible people leave for work in early morning. Interviewers arrive in villages and towns at the time that people were typically at home, either mid-day in villages or very early or very late in towns.

Enumerators used pen/pencil and paper in 2015 and smart phones programmed with Open Data Kit software[23] to record data in 2016. All questionnaires were uploaded daily (or when in a mobile network was available) to a *Google cloud* database for cleaning and analysis; daily updates and results were sent to the national programmes and the provincial health and medical directors.

Data collection in each SA required an average of three-days. The study, from training through analysis and discussion of the results, took three-weeks in Mozambique; four-weeks in the Kasai region of DRC and two-weeks in the Kongo Central province.

### Statistical analysis

LQAS analyses were carried out mainly in Excel so that IU teams could to do their own analysis while the authors used SPSS to the complete analysis and to check IU level results. We weighted district SA data by the district SA population sizes for calculation of coverage and 95% confidence intervals.

## Results

In Mozambique, verified provincial coverages were lower than those reported nationally by 6.5 to 26.1 percentage points ($\bar{x}$ = 17.4, SD = 8.2). In the DRC, verified provincial coverages were -1.6 to 27.5 percentage points ($\bar{x}$ = 10.7, SD = 11.5) lower than those reported (Tables 2 and 3).

**Table 2:** Mozambique districts, populations, sample sizes and study results July 2016*.

Province with Districts	Population	Number of Supervision Areas	Sample Size	Took ALB and IVM	Weighted Verified Coverage (95%CI)(95% CI) Classification DR = 13	Reported Coverage
**Cabo Delgado**	**450,161**	**5**	**95**	**72**	76.9% (± 8.5%)	94%
Moc Praia	110,795	1	19	15	Pass	
Mueda	131,723	2	19	16/14	Pass	
Nangade	73,378	1	19	16	Pass	
Palma	53,576	1	19	11	Fail	
**Niassa**	**331,335**	**4**	**76**	**63**	81.5% (± 9.9%)	88%
Cuamba	249,843	2	38	16/15	Pass	
Maúa	63,755	1	19	15	Pass	
Mecula	17,738	1	19	17	Pass	
**Nampula**	**1,713,489**	**8**	**152**	**101**	65.9% (± 8.3%)	92%
Larde**	89,203	1	19	10	Fail	
Liupo**	82,624	1	19	12	Fail	
Mogincual	106,056	1	19	14	Pass	
Mogovolas	430,083	1	19	16	Pass	
Moma**	375,901	1	19	7	Fail	
Monapo	384,171	1	19	13	Pass	
Muecate	116,887	1	19	14	Pass	
Nacaroa	128,534	1	19	15	Pass	
**Zambezia**	**416,523**	**4**	**76**	**66**	86.8% (± 8.5%)	100%
Inhassunge	103,741	2	38	17/16	Pass	
Mopeia	160,795	1	19	16	Pass	
Namarrói	151,987	1	19	17	Pass	
**Total**	**2,911,478**	**21**	**399**	**302**	**72.2%** **(±1.8%)**	**95%**

*ALB = Albendazole, IVM = Ivermectin, CI = Confidence Interval

** Districts re-treated and the LQAS survey repeated.

**Table 3:** DRC verified coverage of Ivermectin and Albendazole by province in 2015 and 2016*.

Province	Took IVM	Took ALB	Sample Size	Weighted verified coverage (95% CI)	Reported Coverage
Kongo Central (2015)	n.a.	135	171	78.6% (±6.5%)	87%
Kongo Central (2016)	n.a.	174	190	90% (±5%)	97%
Kasai (2016)	79	79	95	82.8% (±7.8%) ALB, IVM	96%
Kasai Central (2016)	89	86	95	94.6% (±3.5%) ALB 95.7% (±3.8%) IVM	93%
Kasai Oriental (2016)	110	114	152	72.8% (±8.2%) ALB 68.5% (±8.4%) IVM	96%

*ALB = Albendazole, IVM = Ivermectin, CI = Confidence Interval

In Mozambique, IVM and ALB were distributed at the same time; therefore, we report one coverage estimate. Out of 21 SA LQAS classifications, four districts failed to reach the minimum coverage target. Nampula province, whose average endemicity is 53% in all its 23 districts, had 65.9% (95%CI: ±8.3%) coverage with three failing districts (Table 2). The remaining failure was in Cabo Delgado province. Two main reasons were given for not taking the medication: absence from home on the day of the campaign (38%), or illness (10%). Lymphoedema (55.6%) and worms (41%) were the main reasons for taking the medication. Only 58 respondents (14.5%) reported mild side effects (dizziness) after taking the medication.

In 2015, the survey coverage of ALB in the Kongo Central province of DRC was 78.6% (95% CI: ±6.5%) vs. 87% reported coverage, with one district failing to achieve the 80% target. A pre-distribution census reached 92% of households contributing to the overestimation of coverage. People not registered in the census were less likely to receive the medication than registered persons. Only 21% of non-registered persons took the medication, versus 84% of those registered. The main reasons for non-registration and not taking the medication were absence from home (50%) and fear of adverse reactions (30%). ALB caused minor side effects in less than 1% of the 573,550 medicated persons reached by the first campaign.

In 2016, districts and province health departments applied lessons learned from the first campaign and survey, with emphasis on transmitting medication safety and ensuring CDDs returned to the houses of the absent, no district in Kongo Central was classified as not reaching the target. Overall, 95% of the eligible population was registered, with 97% reported coverage and 90% (95% CI: ±5%) verified coverage (Table 3). While absence from home was still the main reason for not taking ALB (50%), fear of adverse effects was reduced to 13%, due to improvements in provincial and district supervision, social mobilisation and the lack of serious adverse effects in the previous year.

For Kasai province, the ALB/IVM verified coverage was 82.8% (95%CI: ±7.8%). Coverage of each medication differs indicating that drug distribution took place at two different points in time. All 13 districts and 18 SAs reached their targets, except the district of Lubilanji in the city of Mbuji-Maji which reduced the Kasai Oriental provincial coverage to 72.8% (95%CI:±8.2%), well under the target. (See S1 and S2 Tables for the DRC LQAS results.)

In Kasai province, the main reason for not taking the medication was absence from home during the campaign. Although most interviewees knew the reasons for the campaign, Kasai and Kasai Central provincial residents frequently mentioned onchocerciasis and filariasis, and their consequences, as their main reasons for taking the medication. In Kasai Oriental province, treating worms was the primary incentive for taking the medication.

Both national programmes reviewed the survey data daily and rapidly reacted to the results. In Mozambique, the decision was to treat again all eligible population in the three districts of Nampula province, reinforce training and support of district teams and improve supervision. In a subsequent LQAS assessment after the repeated campaign none of the three districts were classified as not reaching the 80% eligible population coverage target. Cabo Delgado district was not retreated due to lack of medication and funds.

In the DRC, the strategy was to identify and treat individuals not treated during the campaign. Meetings with district and sub-district officers and CDDs were held to review results and treatment registers, identify underperforming sub-districts, reinforce CDD training and supervision, and intensify social mobilization. These actions resulted in the treatment of 12,644 additional people. In a subsequent LQAS survey, previously underperforming sub-districts were classified as reaching the targets for both medications.

## Discussion

The WHO NTD Road Map target is to eliminate LF as a public health problem by 2020. In principle, MDA over a 5-year period should reduce LF transmission to public health elimination targets, but concern is growing that the 2020 target will not be met [4]. WHO 2017 reports indicated that of the 72 endemic countries for LF, 21 of them have completed interventions and are conducting multi-year surveillance to validate elimination, 52 countries require MDA of which 33 need to achieve effective medication coverage, 13 need to treat all endemic districts and achieve 100% geographical coverage and five have yet to start MDA.

Despite this impressive expansion of MDA since 2000 with 7.1 billion treatments administered, delays in programme implementation, issues with compliance and contraindications with some drugs in co-endemic areas mean that elimination will not be met unless MDA interventions are improved and “more than effective” coverage is reached particularly in IU where two medications (IVM+ALB) are used. There are clear examples of this with French Polynesia after three decades, and India, Nigeria and Tanzania after 8–10 years failing to halt transmission[24]. The elimination of *Wuchereria bancrofti* transmission and the ensuing disease and disability in large disease endemic countries such as Mozambique and DRC, which has restricted local capacity and the added complications of Onchocerciasis and Loa co-endemic areas, requires a viable national strategy[25] and a strong health care system. Any strategy must have consistent coordination and efficient operational plans to annually organize and complete national MDAs that distribute one to three medications, depending on the NTDs in the districts[26]. The challenges and costs, including the involvement and assistance of international organizations, are significant.

In Mozambique, the campaigns follow the immunization campaign strategy and takes one week. The 2016 campaign required all national NTD team members and the public health department of the Ministry of Health, mobilization of 114 district teams 312 provincial supervisors and coordinators, 7,320 health care staff and 7,560 community volunteers to treat 16 million people with IVM and ALB.

In the DRC, yearly integrated campaigns distributed drug combinations. In 2016, the national NTD programme reached a geographical coverage of 36% of the endemic districts in need of treatment. This effort included 169 districts and 2,819 sub-district teams, 6,693 health staff and 137,897 CDDs delivering medication to 27 million people door-to-door in line with the Community Directed Treatment with IVM approach[27]. The MDA covered four endemic diseases taking 3-months to complete at the distribution and data collection stages[28].

LQAS surveys helped both countries to identify areas with probable low coverage and to adopt strategies to increase coverage and reach targets. In contrast to a system that relied on administrative data, results were available at the district level before medication was returned to the provincial depots, simplifying the logistics of retreatment. In both countries, LQAS coverage measures aggregated to the provincial level were typically lower than the reported coverage for the majority of provinces revealing gaps in coverage.

In Mozambique a district health system response focused on better informing communities via community leaders of the campaign’s date, reasons for taking the medication, and encouraging people to be present when the mobile teams arrive in the community. Health staff during in the lead-up to and when launching the campaigns focused on the safety of the medication and the lack of serious side-effects to address earlier worries in the population. Reducing absenteeism was also noted in Krentel’s [29] review of 79 papers as this was a common reason for non-compliance. The retreatment strategy emphasized strong supervision to ensure mobile teams returned to homes of absentees while emphasising the need to reach coverage targets.

In DRC, the district and provincial teams with the CDDs reviewed the registers and identified the areas where registration (and distribution) had not reached targets. CDDs and supervisors then targeted specific neighbourhoods and communities to reinforce information about the campaigns date, the benefits of the medication and the possibility of a second chance to take the medication at home or in the CDD’s home; this simple adjustment reduced the workload of CDDs as they often did not need to return twice to the same house. DRC also developed and implemented checklists for each level of supervision, and reinforced training of district and provincial supervisors.

The LQAS verification survey permits a principled approach for identifying and mopping-up priority districts with low coverage and calculating a more accurate provincial coverage. The LQAS data identified that the reported coverage in DRC, uses the registered eligible population rather than the estimated eligible population as the denominator; this resulted in coverage being inflated due to underestimation of the denominator. A similar denominator estimation was recently reported in Benin and Madagascar [30]. The LQAS survey provided the percentage of the eligible registered population that was covered and the direction of the error of the reported coverage. Lessons learned from the 2015 survey were applied by Kongo Central province in 2016. Registration increased from 92% to 95% of the eligible population and all SA achieved targets.

The survey showed that during the first distribution (in 2015) of ALB in Kongo Central, 13% of the population did not take the medication out of fear of adverse effects. This reaction occurred when IVM was administered in *Loa loa* endemic areas in 2005 in Oncho-*Loa* endemic areas of Kongo Central and was associated with encephalitis due to the death of the microfilaria of the *Loa* parasite in people with high levels of microfilaria (>30,000 microfilaria/ml of blood) of the parasite[31]. Subsequently, after identification of co-endemic Loasis and prompt diagnosis, no deaths occurred. However, the population was still afraid of taking medication. The enhanced mobilization in 2016 and the lack of adverse effects in 2015, resulted in less than 5% of non-recipients mentioning adverse effects as a reason for their non-registration and non-intake of ALB.

The main incentive the population in both countries cited for taking the medication was treatment of LF (lymphoedema and hydroceles). This result is consistent with the 2013 survey [29]. The district of Lubilanji, in the DRC, was an exception where most respondents indicated that they believed the medication was for “worms.” Results like these help to shape mop-up and subsequent MDA to reach recurrent coverage targets.

### Limitations

This study has limitations. The LQAS method would have incorrectly classified districts near to but not achieving the 80% eligible population target, as having reached the target. However, a conventional cluster survey may have experienced a similar problem because a 95% confidence interval can have a range of values that are both above and below the coverage target. This assessment interviewed individuals who were present in the village. This could have led to bias as individuals who did not take medication during the MDA would not have been included in the sampling. This limitation is one that is shared by other survey methods.

### Conclusion

This study demonstrated the usefulness of LQAS for routine monitoring and evaluation of national MDA programmes to produce rapid improvements in traditionally weak health systems. These results are consistent with the value of LQAS in other non-MDA programmes[32]. The important contribution for NTD control was to rapidly identify areas likely to have low coverage where transmission would therefore, continue. It provided accurate local evidence permitting immediate feedback to identify service delivery problems[33] that national programmes can use corrective action.

## Supporting information

S1 Table DRC populations, supervision areas and samples collected and classification of districts by intake of Albendazole in the province of Kongo Central in 2015 and 2016. (DOCX)

S2 Table DRC populations, supervision areas and samples collected in Kasai Region in 2016. (DOCX)
