# Supplementary material for: Improved assessment of mass drug administration and health district management performance to eliminate lymphatic filariasis
Source: PLoS Negl Trop Dis. 2019 Jul 5;13(7):e0007337. doi: 10.1371/journal.pntd.0007337 (PMC6636779; doi:10.1371/journal.pntd.0007337)
Supplement: S1 Table — (DOCX) [file pntd.0007337.s001.docx]

Table S1. DRC populations, supervision areas and samples collected and classification of districts by intake of Albendazole in the province of Kongo Central in 2015 and 2016

| **2016 Zone de Santé**  **(District)** | **Estimated Population** | **Number of Supervision areas 2015 (2016)** | **2015** | | | **2016** | | |
| --- | --- | --- | --- | --- | --- | --- | --- | --- |
|  |  |  | **Sample Size** | **Took ALB** | **Classification***  **DR=13**  **Weighted Coverage (+95%CI)** | **Sample Size** | **Took ALB** | **Classification* DR=13**  **Weighted Coverage (+95%CI)** |
| Boma | 204,167 | 1 | 19 | 15 | Pass | 19 | 16 | Pass |
| Boma Bungu | 85,960 | 1 | 19 | 17 | Pass | 19 | 18 | Pass |
| Kitona | 79,739 | 1 | 19 | 16 | Pass | 19 | 18 | Pass |
| Kizu | 67,213 | 1 | 19 | 16 | Pass | 19 | 17 | Pass |
| Kuimba** | 101,653 | 1  (2 in 2016) | 19 | 11 | Fail | 38 | 19,18 | Pass |
| Muanda | 143,486 | 1 | 19 | 16 | Pass | 19 | 18 | Pass |
| Nzanza | 147,056 | 1 | 19 | 14 | Pass | 19 | 16 | Pass |
| Tshela | 96,539 | 1 | 19 | 16 | Pass | 19 | 16 | Pass |
| Vaku | 77,000 | 1 | 19 | 14 | Pass | 19 | 18 | Pass |
| **Total** | **1,002,813** | **9 (10)** | **171** | **135** | **78.6%**  **(+6.5%)** | **190** | **174** | **90%**  **(+ 5%)** |

*ALB=Albendazole, IVM=Ivermectin, CI=Confidence Interval

**Kuimba failed to reach targets in 2015. The provincial programme suggested differences in the district, which was divided into two subdistricts in 2016.
