# Supplementary material for: Improved assessment of mass drug administration and health district management performance to eliminate lymphatic filariasis
Source: PLoS Negl Trop Dis. 2019 Jul 5;13(7):e0007337. doi: 10.1371/journal.pntd.0007337 (PMC6636779; doi:10.1371/journal.pntd.0007337)
Supplement: S2 Table — (DOCX) [file pntd.0007337.s002.docx]

**Table S2. DRC populations, supervision areas and samples collected in Kasai Region in 2016.***

| **Province** | **Zone de Santé (District)** | **Estimated Population** | **Number of Supervision Areas** | **Sample**  **Size** | **Albendazole** | | **Ivermectin** | |
| --- | --- | --- | --- | --- | --- | --- | --- | --- |
|  |  |  |  |  | **Took ALB** | **Classification DR=13**  **Weighted Coverage (+95%CI)** | **Took IVM** | **Classification DR=13**  **Weighted Coverage (+95%CI)** |
| **Kasai** | Kamonya 1  Kamonya 2 | 426,385 | 2 | 38 | 16, 17 | Pass  Pass | 16,  17 | Pass  Pass |
|  | Luebo | 231,671 | 1 | 19 | 14 | Pass | 14 | Pass |
|  | Mutenna | 129,436 | 1 | 19 | 17 | Pass | 17 | Pass |
|  | Ndjoko Punda | 115,262 | 1 | 19 | 15 | Pass | 15 | Pass |
|  | Total | 902,754 | 5 | 95 | 79 | 82.8%  (+7.8%) | 79 | 82.8%  (+8%) |
| **Kasai Central** | Kananga 1  Kananga 2 | 215,563 | 2 | 38 | 15, 17 | Pass | 17, 18 | Pass |
|  | Katende | 35,758 | 1 | 19 | 16 | Pass | 17 | Pass |
|  | Lukonga | 278,636 | 1 | 19 | 19 | Pass | 19 | Pass |
|  | Mutoto | 201,424 | 1 | 19 | 19 | Pass | 18 | Pass |
|  | Total | 731,381 | 5 | 95 | 86 | 94.6%  (+3.5%) | 89 | 95.7%  (+3.8%) |
| **Kasai Oriental** | Bibanga 1  Bibanga 2  Bibanga 3 | 142,101 | 3 | 57 | 16,  14,  15 | Pass | 16, 14,  15 | Pass |
|  | Bonzola | 204,695 | 1 | 19 | 15 | Pass | 13 | Pass |
|  | Lubilanji | 265,652 | 1 | 19 | 12 | Fail | 10 | Fail |
|  | Mpokolo 1  Mpokolo 2 | 316,013 | 2 | 38 | 14, 14 | Pass | 13,  14 | Pass |
|  | Tshitenge | 187,982 | 1 | 19 | 14 | Pass | 15 | Pass |
|  | Total | 1,116,443 | 8 | 152 | 114 | 72.8%  (+7%) | 110 | 68.5%  (+8.4%) |

*ALB=Albendazole, IVM=Ivermectin, CI=Confidence Interval
